# Pea Grain Protein Content Across Italian Environments: Genetic Relationship With Grain Yield, and Opportunities for Genome-Enabled Selection for Protein Yield

**DOI:** 10.3389/fpls.2021.718713

**Published:** 2022-01-03

**Authors:** Margherita Crosta, Nelson Nazzicari, Barbara Ferrari, Luciano Pecetti, Luigi Russi, Massimo Romani, Giovanni Cabassi, Daniele Cavalli, Adriano Marocco, Paolo Annicchiarico

**Affiliations:** ^1^Council for Agricultural Research and Economics (CREA), Research Centre for Animal Production and Aquaculture, Lodi, Italy; ^2^Department of Agricultural, Food and Environmental Science, University of Perugia, Perugia, Italy; ^3^Department of Sustainable Crop Production, Catholic University of Sacred Heart, Piacenza, Italy

**Keywords:** crop quality, crude protein yield, genetic variation, genomic selection, genotype × environment interaction, grain yield, inter-population prediction, *Pisum sativum*

## Abstract

Wider pea (*Pisum sativum* L.) cultivation has great interest for European agriculture, owing to its favorable environmental impact and provision of high-protein feedstuff. This work aimed to investigate the extent of genotype × environment interaction (GEI), genetically based trade-offs and polygenic control for crude protein content and grain yield of pea targeted to Italian environments, and to assess the efficiency of genomic selection (GS) as an alternative to phenotypic selection (PS) to increase protein yield per unit area. Some 306 genotypes belonging to three connected recombinant inbred line (RIL) populations derived from paired crosses between elite cultivars were genotyped through genotyping-by-sequencing and phenotyped for grain yield and protein content on a dry matter basis in three autumn-sown environments of northern or central Italy. Line variation for mean protein content ranged from 21.7 to 26.6%. Purely genetic effects, compared with GEI effects, were over two-fold larger for protein content, and over 2-fold smaller for grain and protein yield per unit area. Grain yield and protein content exhibited no inverse genetic correlation. A genome-wide association study revealed a definite polygenic control not only for grain yield but also for protein content, with small amounts of trait variation accounted for by individual loci. On average, the GS predictive ability for individual RIL populations based on the rrBLUP model (which was selected out of four tested models) using by turns two environments for selection and one for validation was moderately high for protein content (0.53) and moderate for grain yield (0.40) and protein yield (0.41). These values were about halved for inter-environment, inter-population predictions using one RIL population for model construction to predict data of the other populations. The comparison between GS and PS for protein yield based on predicted gains per unit time and similar evaluation costs indicated an advantage of GS for model construction including the target RIL population and, in case of multi-year PS, even for model training based on data of a non-target population. In conclusion, protein content is less challenging than grain yield for phenotypic or genome-enabled improvement, and GS is promising for the simultaneous improvement of both traits.

## Introduction

Greater cultivation of grain and forage legumes is a priority for European agriculture to enhance its sustainability while reducing its huge deficit for high-protein feedstuff (Pilorgé and Muel, 2016). The positive impact of legume cultivation concerns energy and resource use efficiency, greenhouse gas emissions, nitrogen biogeochemical fluxes, and agricultural biodiversity (Nemecek et al., 2008; Billen et al., 2014; Foyer et al., 2016). Grain legume cultivation has been promoted by the European Common Agricultural Policy through various supporting measures, but its expansion is hindered by substantial yield and profitability gap with respect to major cereal crops (Schreuder and De Visser, 2014).

Field pea (*Pisum sativum* L.), compared with other cool-season grain legumes, tended to display greater yield potential in Western (Carrouée et al., 2003) and Southern Europe (Annicchiarico, 2008) and moderately good rate of genetic yield progress (Annicchiarico, 2017). However, the grain protein content of commercial cultivars is only moderate (usually in the range 22–26% on a dry-matter basis), and increased protein content represents a major breeding objective (Duc et al., 2015). The reported range of variation for crude protein content among relatively large sets of breeding lines or modern cultivars was fairly inconsistent. It was around 3.5% in Tar’an et al. (2004) and Burstin et al. (2007), 8% in Cousin et al. (1985), and close to 10% in Jha et al. (2015) and Ferrari et al. (2016). The range exceeded 10% in a recombinant inbred line (RIL) population issued by parents with contrasting protein content (Irzykowska and Wolko, 2004) and a world collection of ecotypes and old cultivars (Annicchiarico et al., 2017), while exceeding 16% in germplasm accessions from regional or global collections (Blixt, 1978) and material encompassing modern lines and wild relatives (Tzitzikas et al., 2006).

Genotype × environment interaction (GEI) effects for grain protein content have not been thoroughly investigated. They were modest and/or nonsignificant in Matthews and Arthur (1985) and in Krajewski et al. (2012), while being significant but with no estimation of their size (e.g., in terms of variance component relative to purely genetic effects or genetic correlation across test environments) in Burstin et al. (2007). In contrast, outstanding GEI was repeatedly observed for grain yield, particularly across environments of southern Europe (Annicchiarico and Iannucci, 2008; Iglesias-García et al., 2017; Pecetti et al., 2019), where it was more affected by year-to-year climatic variation than by geographical distance (e.g., within the Italian target region).

Since the selection for higher grain protein content is likely to be performed concurrently with selection for higher crop yield, the genetic correlation between these characters has crucial importance for pea breeding. However, earlier studies assessed only phenotypic correlations, and their indications were inconsistent. An inverse correlation around 0.4 was found by Tar’an et al. (2004) and by Krajewski et al. (2012), whereas no correlation was reported by Cousin et al. (1985) and Bărbieru (2021). In a large study by Klein et al. (2020), the phenotypic correlation between grain protein content and seed weight per plant was slightly negative overall (*r* = −0.11) but varied largely across populations and environments.

Several molecular studies reported quantitative trait loci (QTL) for pea grain protein content (Irzykowska and Wolko, 2004; Tar’an et al., 2004; Burstin et al., 2007; Bourion et al., 2010; Krajewski et al., 2012; Klein et al., 2014, 2020; Jha et al., 2015; Gali et al., 2018, 2019). Their results indicated modest trait variation accounted for by the vast majority of individual QTL, as well as fairly widespread inconsistency of QTL across test environments. The only moderate number of markers these studies were based upon (ranging from 106 to 680) limited a thorough investigation of QTL and trait genetic architecture. However, the polygenic control that they suggested challenged the adoption of marker-assisted selection (MAS) for protein content. Genomic selection (GS), by which breeding values for polygenic traits are predicted by a statistical model constructed from genome-wide marker information (Meuwissen et al., 2001), can be more convenient than MAS in this situation (Bernardo and Yu, 2007). However, this selection strategy requires the availability of high numbers of molecular markers spread across the genome, as made possible for a reasonably low cost by a high-throughput genotyping technique such as genotyping-by-sequencing (GBS; Elshire et al., 2011). In addition, high marker number increases the ability of genome-wide association studies (GWAS) to unveil the trait genetic architecture and to identify relevant genomic regions, especially for species with a sequenced genome such as pea (Kreplak et al., 2019). Genome-enabled predictions proved sufficiently accurate to encourage GS as a partial substitute for PS for pea grain yield under moisture-favorable (Annicchiarico et al., 2019) and severely drought-prone regions (Annicchiarico et al., 2020), but no report is available on its potential value for improving pea protein content or pea protein yield per unit area.

An earlier study by Annicchiarico et al. (2019) reported on GEI extent across Italian environments, genome-enabled predictions and GS predicted efficiency relative to phenotypic selection (PS) for grain yield of pea breeding lines belonging to three RIL populations issued by connected crosses between elite parent cultivars. That work assessed not only intra-population genome-based predictions (where GS model training includes the RIL population targeted by selection) but also inter-population predictions (where other non-target, connected RIL populations were used for GS model training). This study, based on the same plant material and testing environments, added to results by Annicchiarico et al. (2019) with the aim of assessing (a) the extent of GEI for grain crude protein content, (b) the genetic correlation between grain yield and protein content, (c) the extent of polygenic control for protein content and grain yield and major relevant genomic areas for these traits as suggested by GWAS, and (d) the predictive ability of GS for improving crude protein yield and its predicted efficiency relative to PS, envisaging both intra-population and inter-population prediction scenarios.

## Materials and Methods

### Plant Material

This study encompassed the same plant material and test environments described in Annicchiarico et al. (2019) for the phenotyping of grain yield responses. In brief, it included 306 genotypes belonging to three RIL populations issued from connected crosses between three cultivars, i.e., the European cultivars Attika and Isard, and the Australian cultivar Kaspa, which featured high and stable grain yield across Italian climatically contrasting environments in a previous assessment of a large number of modern cultivars (Annicchiarico, 2005; Annicchiarico and Iannucci, 2008). In particular, the number of lines per cross was 102 for Attika × Isard (hereafter A × I), 100 for Kaspa × Attika (K × A), and 104 for Kaspa × Isard (K × I). Samples of DNA for genotyping were extracted from four F_6_ plants per line grown in a non-heated glasshouse, while phenotyping was carried out on individuals obtained after one additional generation of multiplication. Phenotyping experiments also included the parental cultivars and the cultivar Spacial, which was used as a control cultivar because of its high yielding ability across Italian environments (Pecetti et al., 2019).

### Phenotyping

The set of 310 genotypes was autumn-sown in three rain-fed test environments identified hereafter by the combination of location and growing season as Lodi 2013–2014, Lodi 2014–2015 and Perugia 2013–2014. Lodi (45°19′N, 9°30′E) is located in northern Italy and is characterized by a subcontinental climate, whereas Perugia (43°06′N, 12°23′E) features the cool Mediterranean climate typical of central Italy and inland southern Italy (Supplementary Table 1). One test site (Lodi) included two cropping years, to widen the assessment of GEI, which, for pea in Italian environments, is mainly affected by year-to-year variation (Annicchiarico and Iannucci, 2008; Pecetti et al., 2019). Crop management contributed to widening the environment variation, since Lodi 2013–2014 and Perugia 2013–2014 underwent an organic management, whereas Lodi 2014–2015 was managed conventionally. Each experiment was designed as a randomized complete block with three replicates. Additional details regarding plot size, experiment set up and management, and grain yield assessment can be found in Annicchiarico et al. (2019). Grain protein content was determined through near-infrared spectroscopy (NIRS). Before analysis, a random sample of 100 g of dry seeds for each plot was ground by a cutting mill (Pulverisette 19, Fritsch GmbH, Germany) equipped with a sieve of 1 mm mesh. Flour samples were analyzed in the 1,000–2,500 spectral range by employing a Nirflex 500 spectrometer (Büchi, Italy). An *ad-hoc* calibration using PLS Toolbox 8.9 (Eigenvector Research Inc.) was developed using the protein content of 262 flour samples determined *via* chemical analysis as reference measurements. These samples were selected from the whole experimental set according to a Kennard Stone multivariate design, while selecting 40 additional independent samples for validation. For chemical analysis, flour samples were further ground by a MM 400 mixer mill (Retsch Gmbh & Co., Germany), and total N was determined in duplicate by dry combustion (Dumas method) using a ThermoQuest NA1500 elemental analyzer (Carlo Erba, Milano, Italy) and atropine as a standard. A multivariate filtering (external parameter orthogonalization) was applied as pre-processing to the spectra to reduce the bias between years and locations. The developed calibration model for the prediction of N content showed a standard error of prediction (SEP) of 0.16 g N/100 g (*R*^2^ = 0.94) that was comparable with the chemical analysis error of 0.15 g N/100 g. Seed protein content was calculated by multiplying the NIRS-estimated N content by 6.25. Dry-weight crude protein yield per unit area was computed on a plot basis by multiplying dry grain yield by grain protein content plot values.

### Statistical Analysis of Phenotypic Data

The following analyses concerned grain crude protein content, grain yield, and crude protein yield per unit area of the lines belonging to the RIL populations. An analysis of variance (ANOVA) including the random factors genotype and block was performed for each RIL population in each environment to assess the significance of the within-population variation and its extent as genetic coefficient of variation computed as:


$$CV_{g}=\left(S_{G}/m\right)\times 100$$

where *S_G_* is the square root of the genotype component of variance ($S_{G}^{2}$), and *m* is the trait mean value. Here and in the following analyses, variance components were estimated by a restricted maximum likelihood method. An ANOVA including the fixed factor environment and the random factors genotype and block within environment aimed to test the significance of environmental, genotypic, and GEI effects, using the same model to estimate variance components relative to genotype ($S_{G}^{2}$) and GEI ($S_{GE}^{2}$). A further ANOVA included the fixed factor environment and the random factors RIL population, genotype within RIL population, and block within environment, according to the following model:


$$Y_{kijr}=m+R_{k}+G_{i}\left(R_{k}\right)+E_{j}+B_{r}\left(E_{j}\right)+R_{k}E_{j}+G_{i}\left(R_{k}\right)E_{j}+e_{kijr}$$

where *Y_kijr_* is the observed response of the genotype *i* belonging to the RIL population *k* in the block *r* of the environment *j*, *m* is the grand mean, and *R_k_*, *G_i_*, *E_j_*, and *B_r_* correspond to RIL population, genotype, environment and block effects, respectively. This ANOVA model aimed to test the significance of relevant effects and to estimate variance components relative to RIL population ($S_{R}^{2}$), genotype within population ($S_{G\left(R\right)}^{2}$), RIL population × environment interaction ($S_{RE}^{2}$), and genotype within population × environment interaction $(S_{G\left(R\right)E}^{2}$). RIL populations were compared for mean trait value in each environment by an ANOVA including population as fixed factor and genotype within population and block as random factors. The extent of GEI across pairs of environments represented by (a) different growing seasons in the same environment (2013–2014 and 2014–2015 in Lodi) or (b) different locations in the same cropping season (Lodi and Perugia in 2013–2014) was assessed in terms of genetic correlation for genotype response according to the formula (Basford et al., 2004):


$$r_{g}=r/\left(H_{1}H_{2}\right)$$

where *r* is the phenotypic correlation for genotype values across the relevant pairs of environments, and *H_1_* and *H_2_* are the square root of the broad-sense heritability (*H^2^*) calculated on a genotype mean basis in each environment from variance components for genotype ($S_{G}^{2}$) and experimental error ($S_{e}^{2}$) and *n* experiment replications as:


$$H^{2}=S_{G}^{2}/\left(S_{G}^{2}+S_{e}^{2}/n\right).$$

Broad-sense heritability values were also used to calculate best linear unbiased predictions (BLUP) values according to DeLacy et al. (1996) that served as phenotypic data for GS and GWAS analyses. The genetic correlation between grain yield and grain protein content was estimated for each environment according to Piepho and Mohring (2011). We assessed the impact on protein yield of each of its component traits (grain yield and grain protein content) in each environment by Pearson’s phenotypic correlation.

An ANOVA limited to the parent cultivars and the reference cultivar Spacial that held genotype as fixed factor and block as random factor was performed for each environment to compare the four cultivars. An additional ANOVA including all genotypes (lines and cultivars) that held genotype and environment as fixed factors and block as random factor was carried out to assess the number of inbred lines that outyielded the control variety Spacial and the top-performing parent cultivar for the trait of greatest practical interest, namely protein yield per unit area.

All statistical analyses were carried out using SAS/STAT® or R studio software.

### DNA Isolation, GBS Library Construction, and Sequencing

Information on DNA isolation and GBS can be found in Annicchiarico et al. (2017). Raw reads for library construction were demultiplexed using axe demultiplexer (Murray and Borevitz, 2018). Trimming for restriction enzyme remnants, alignment on reference genome and SNP calling were performed using the dDocent pipeline (Puritz et al., 2014), employing the *Pisum sativum* L. reference genome version 1a (Kreplak et al., 2019)^1^. The SNP calling procedure differed from that used in the earlier study for grain yield, where it relied on a mock genome (Annicchiarico et al., 2019). The final genotype matrix, in the form of a vcf file, was filtered for quality using the vcftool software (Danecek et al., 2011) with parameters –minQ 30 –max-non-ref-af 1 –non-ref-af 0.001. The resulting data set was filtered for increasing levels of allowed missing values, excluding markers showing a missing rate across genotypes greater than a fixed threshold of 5, 10, 15, 20, and 30%. Markers that were monomorphic or with minor allele frequency < 5% were removed. After marker filtering, samples were also filtered for growing missing rate levels, represented by thresholds of 10, 25, and 50%. Following Nazzicari et al. (2016), we estimated missing data by Random Forest imputation through the R package MissForest (Stekhoven and Bühlmann, 2012) with the configuration ntree = 100, maxiter = 10, defining genotypes as categorical data (factors).

### Genomic Regression Models and Data Configurations

We assessed the intra-population, inter-environment prediction scenario by performing model training on genotype values of 90% of the lines averaged across two environments and validation on the remaining 10% of lines in the third environment with 10 repetitions of this 10-fold stratified cross-validation scheme, using by turns all possible combinations of training and validation environments. Each training and validation set contained an equal proportion of lines from each of the three RIL populations. Predictive ability (computed as Pearson’s correlation between the observed phenotypic values and those predicted by GS) was assessed separately for each RIL population, to avoid bias due to different population means. Results were averaged across repetitions, sets of training environments and RIL populations. This analysis was initially exploited to define the optimal thresholds of missing data per marker (mpm) and missing data per sample (mps) by employing the Ridge regression BLUP (rrBLUP) model (Meuwissen et al., 2001), which combined high computation ability with good prediction ability in early studies (Annicchiarico et al., 2019, 2020). We envisaged intra-population, inter-environment predictions according to four possible GS models, namely, rrBLUP, Bayesian C, Bayesian A, and Bayesian Lasso (Meuwissen et al., 2001; Park and Casella, 2008). The rrBLUP model assumes that marker effects have a common variance, which makes it more suitable for traits controlled by a large number of QTL with a small effect, whereas Bayesian models assume relatively few markers with large effects and allow, therefore, markers to have different effects and variances (Wang et al., 2018). Because of its good predictive ability, rrBLUP was selected for assessing inter-population, inter-environment predictions. In this case, model training was performed on data averaged across two environments of a single RIL population, assessing the predictive ability on data of each of the other two RIL populations in the remaining environment. All populations and pairs of environments were used by turns for model training, averaging the results across training sets. Regression models, cross-validation, and predictive ability estimations were all computed through the R package GROAN (Nazzicari and Biscarini, 2017).

### Comparison of Genomic vs. Phenotypic Selection

The correlation of phenotypic data in one (validation) environment with either phenotypic data averaged across the other two (selection) environments or GS-based breeding values obtained from GS model training based on the same data, averaging the results across all possible environment combinations and RIL populations, provided a preliminary assessment of phenotypic vs. genomic predictions. This comparison aimed to assess the possible loss (or gain) of predictive ability derived from GS modelling of phenotypic data relative to that of phenotypic data themselves. In this case, all the genotypes were used for GS model construction.

A comparison of GS vs. PS in terms of selection efficiency for future selection activities taking account of possible differences in selection cycle duration and selection costs was carried out for crude protein yield per unit area, considered as the trait of greatest practical interest. As in earlier analyses, GS hypothesized two training environments (as reasonable in the presence of sizeable GEI) and one validation environment with all environments acting by turns as training or validation, envisaging the two scenarios of intra-population, inter-environment prediction and inter-population, inter-environment prediction. Predictive ability (*r_ab_*) values averaged across RIL populations and all possible sets of training environments were used to estimate GS model accuracy (*r_Ac_*) values according to Lorenz et al. (2011) as: *r_Ac_* = *r_ab_*/*H*, where *H* is the square root of the broad-sense heritability on a genotype mean basis in the validation environment estimated as described earlier. The mean value of *r_Ac_* across RIL populations and validation cycles was imputed in the following formula for estimation of the expected genetic gain per selection cycle from GS (Heffner et al., 2010):


$$\Delta G_{G}=i_{G}\;r_{\mathrm{Ac}}\;s_{A}$$

where *i_G_* = standardized selection differential for GS, and *s_A_* = standard deviation of breeding values. We computed the expected genetic gain per year as:


ΔGG'=iGrAcsA/tG

where *t_G_* = duration in years of one GS cycle, which was set to 0.5 under the hypothesis of two possible selection cycles per year for GS (one off-season and one ordinary).

The expected genetic gain per year from PS is (Falconer, 1989):


ΔGP'=iPHsA/tP

where *i_P_* = standardized selection differential for PS, *t_P_* = duration in years of one PS cycle, and *H* = square root of the broad-sense heritability on a genotype mean basis across the experiments hypothesized for selection, and *s_A_* corresponding to previous notation. We hypothesized two selection experiments, each with three replications, accommodated either at two sites in the same year (implying *t_P_* = 1) or in two years at the same or different sites (implying *t_P_* = 2). For each RIL population, we estimated the broad-sense heritability on a genotype mean basis across each of the three possible pairs of selection environments through the formula:


$$H^{2}=S_{G}^{2}/\left(S_{G}^{2}+S_{GE}^{2}/e+S_{e}^{2}/e\;n\right)$$

where $S_{G}^{2}$, $S_{GE}^{2}\;$and $S_{e}^{2}$ are the genotypic, GEI and pooled experiment error components of variance, respectively, and *e* and *n* are numbers of environments and experiment replications, respectively.

From the formulae above, a comparison of GS vs. PS in terms of predicted genetic gain per year for same overall costs equates to comparing (*i_G_ r_Ac_*/*t_G_*) vs. (*i_P_ H*/*t_P_*), considering the impact on *i_G_* and *i_P_* values of different evaluation cost per genotype of GS and PS. These costs were estimated equal to € 220 for both the outlined PS scenarios, while amounting to approximately € 60 for GS. The hypothetical availability of a fixed budget would imply the possibility to evaluate 3.7 times more genotypes by GS relative to PS. For a large number of lines, the ratio of *i_G_* to *i_P_* would be (2.309/1.755) = 1.316 for a selected fraction of 2.7% for GS and 10% for PS, and (2.023/1.400) = 1.445 for selected fractions of 5.5% for GS and 20% for PS (Falconer, 1989). We decided to adopt an intermediate ratio, namely, $i_{G}$ = 1.381 $i_{P}$.

### Genome-Wide Association Study

For grain yield and protein content we performed a GWAS using the R package statgenGWAS (Van Rossum and Kruijer, 2020). The genotype matrix was used to compute a square kinship matrix (Astle and Balding, 2009), which was employed as covariance matrix in a Generalized Least Squares model to estimate the marker effects and the corresponding values of *p*. The first 10 components of a principal component analysis were included in the GWAS, to account for population structure. The visual inspection of quantile-quantile plots comparing the distribution of trait-marker association scores with a normal distribution expected in case of no significant association (Supplementary Figure 2) confirmed for both traits a convenient accounting of population structure. Together with the values of *p*, we computed the percentage of explained phenotypic variance for each marker (Shim et al., 2015). We envisaged two methods to assess the statistical significance at *p* < 0.05 of trait-marker associations, namely: (a) the Bonferroni correction method, which is known to be overly conservative (Storey and Tibshirani, 2003; Kaler and Purcell, 2019); and (b) the False Discovery Rate (Benjamini and Hochberg, 1995), which can provide a more balanced control of the combination of Type I and Type II error rates (Brzyski et al., 2017; Kaler and Purcell, 2019). When the False Discovery Rate threshold was undefined, we investigated the top-performing markers under the caveat of weaker evidence. For significant markers, we computed linkage disequilibrium (LD) in the form of allelic correlation *R*^2^. Pairs of markers showing an *R*^2^ larger than 0.8 were considered as belonging to the same genetic locus.

## Results

### Phenotypic Variation, Genotype × Environment Interaction and Trait Interrelationships

Grain yield results given in Annicchiarico et al. (2019) are reported again in this study as a reference and to highlight major differences between grain yield and protein content for phenotypic variation patterns or ability of genome-enabled models to predict phenotypic variation. On average, the organically-managed environment of Lodi 2013–2014 featured higher grain yield, grain protein content and protein yield per unit area than the conventionally-managed environment of Lodi 2014–2015 (Table 1), along with more favorable climatic conditions as provided by a milder and wetter winter (Supplementary Table 1). Perugia showed intermediate grain protein content, but lowest protein yield caused by definitely lower grain yield than the other environments (Table 1). Its grain yield response, which could not be related to unfavorable climatic conditions (Supplementary Table 1), was probably due to strong weed competition (Annicchiarico et al., 2019).

**Table 1:** Trait mean value in three test environments of 306 pea inbred lines belonging to three connected RIL populations.

Trait	Lodi 2013-14^a^	Lodi 2014-15^b^	Perugia 2013-14^a^	Standard error of means^b^
Yield (t/ha)^c^	6.31^a^	4.59^b^	2.90^c^	0.35
Protein content (%)	25.32^a^	23.22^c^	24.26^b^	0.15
Protein yield (t/ha)	1.60^a^	1.07^b^	0.70^c^	0.09

Row means followed by different letter differ at p < 0.05. Error degrees of freedom for standard error: 6.

The range of variation for mean values of the 306 inbred lines across environments was 21.7–26.6% for protein content, 1.79–7.77 t/ha for grain yield, and 0.46–1.95 t/ha for protein yield. Various lines outperformed the parent cultivars with the highest trait value for grain yield or grain protein content (for which the top-performing line was Kaspa with 25.5% mean protein content: Supplementary Table 2). The set of inbred lines included highly valuable germplasm for protein yield not only with respect to the parent lines but also compared with the elite commercial variety Spacial. Indeed, six inbred lines outperformed Spacial, and nine outperformed the top-performing parent cultivar (Isard), based on mean comparisons at *p* < 0.05 for protein yield over environments.

The differences among RIL populations for grain protein content in each environment were moderate and mostly not significant (Table 2). There were environment-specific differences among populations for protein yield that reflected those for grain yield, leading for example the population K × A to be lower yielding than K × I for grain and protein yield in Perugia 2013–2014 and Lodi 2014–2015 while performing comparably in Lodi 2013–2014. The ANOVAs indicated the occurrence of differences in RIL population mean value for most traits and environments (Table 2), as well as RIL population × environment interaction for all traits (*p* < 0.01; Table 3). The trend of the RIL population K × I towards top-performing response for grain yield and protein content across environments (Table 2) agreed with the trend of its parental lines Kaspa and Isard towards greater grain yield than the third parent line (Attika) and with the greater protein content of Kaspa relative to the other parent lines (Supplementary Table 2).

**Table 2:** Mean value and genetic coefficient of variation of three traits measured in three test environments on pea lines of three RIL populations derived from connected crosses (A × I, 102 lines; K × A, 100 lines; K × I, 104 lines).

Trait	Environment	Mean value^a^				*CV_g_* (%)^b^		
		A × I	K × A	K × I	Standard error of means^c^	A × I	K × A	K × I
Yield (t/ha)	Lodi 2013–14	5.99^a^	6.33^a^	6.54^a^	0.14	10.1	17.5	18.2
	Lodi 2014–15	5.80^a^	2.52^b^	5.78^a^	0.18	28.0	51.3	33.0
	Perugia 2013–14	2.61^b^	2.77^b^	3.31^a^	0.08	24.8	20.7	14.8
Protein content (%)	Lodi 2013–14	24.72^b^	25.55^a^	25.69^a^	0.10	3.7	3.9	3.3
	Lodi 2014–15	23.23^a^^,^^b^	23.03^b^	23.37^a^	0.10	3.9	3.6	3.9
	Perugia 2013–14	23.29^b^	24.82^a^	24.68^a^	0.11	3.9	4.5	3.4
Protein yield (t/ha)	Lodi 2013–14	1.48^b^	1.62^a^	1.68^a^	0.03	11.1	18.0	18.5
	Lodi 2014–15	1.34^a^	0.58^b^	1.35^a^	0.04	30.6	53.5	34.0
	Perugia 2013–14	0.61^c^	0.69^b^	0.82^a^	0.02	25.6	21.8	14.3

a *Row means followed by different letter differ at p < 0.05*.

b CV_g_ *=* $S_{G}^{2}$ */*m, where *m* = trait mean value. Relevant variance different from zero at *p* < 0.01.

c *Error degrees of freedom: 303*.

**Table 3:** Components of variance relative to genotype ($S_{G}^{2}$), genotype × environment interaction ($S_{GE}^{2}$), RIL population ($S_{R}^{2}$), genotype within RIL population ($S_{G\left(R\right)}^{2}$), RIL population × environment interaction ($S_{RE}^{2}$), and genotype within RIL population × environment interaction ($S_{G\left(R\right)E}^{2}$) for three traits in three test environments of 306 pea lines belonging to three connected RIL populations.

Trait	Analysis without RIL population factor			Analysis with RIL population factor			
	$S_{G}^{2}$	$S_{GE}^{2}$	$S_{G}^{2}/S_{GE}^{2}$	$S_{R}^{2}$	$S_{G(R)}^{2}$	$S_{RE}^{2}$	$S_{G(R)E}^{2}$
Yield (t/ha)	0.575^**^	1.435^**^	0.401^**^	0.080^**^	0.520^**^	1.121^**^	0.693^**^
Protein content (%)	0.724^**^	0.302^**^	2.393^**^	0.131^**^	0.637^**^	0.199^**^	0.167^**^
Protein yield (t/ha)	0.036^**^	0.085^**^	0.422^**^	0.003^**^	0.034^**^	0.068^**^	0.040^**^

** *Relevant variance different from zero at p < 0.01*.

Genetic coefficients of variation (*CV_g_*) reported in Table 2 provided information on within-population genetic variability. Significant variation was found for all traits in each environment. *CV_g_* values were definitely smaller for grain protein content than for grain or protein yield, with the latter two traits displaying similar values for specific RIL population-environment combinations (Table 2). As reported in Annicchiarico et al. (2019), the greater within-population variation for grain and protein yield observed in Lodi 2014–2015 was due to variation in winter survival, which was enhanced in this environment by lower winter temperatures relative to the other environments (Supplementary Table 1). The assessment of variance components for pooled genotypes of the RIL populations revealed over two-fold larger purely genetic effects ($S_{G}^{2})$ relative to GEI effects ($S_{GE}^{2}$) for grain protein content, in contrast with the over two-fold larger GEI effects relative to purely genetic effects that was observed for grain and protein yield (Table 3). The estimation of variance components by the ANOVA model including also the RIL population factor highlighted for all traits the occurrence of much greater within-population than among-population genetic variation across environments, whereas GEI was somewhat more affected by RIL population × environment interaction than by genotype within population × environment interaction (Table 3). Genetic correlations for line values of grain and protein yield across environments indicated much lower correlation, hence much greater GEI, across cropping years in Lodi than across locations in 2013–2014 (Table 4), thereby confirming the greater extent of genotype × year interaction over genotype × location interaction in this target region. GEI patterns for line values of grain yield were thoroughly investigated in an earlier study (Annicchiarico et al., 2019). Albeit statistically significant, GEI effects for grain protein content did not imply marked inconsistency of genotype responses across years or locations on the ground of the fairly high genetic correlation values (*r_g_* ≥ 0.73; Table 4). The joint effect of genotypic and GEI variation led to much greater broad-sense heritability on a genotype mean basis, averaged over environments and RIL populations, for grain protein content (*H*^2^ = 0.82) than for grain and protein yield (*H*^2^ = 0.52 and *H*^2^ = 0.54, respectively).

**Table 4:** Significance of genotype × environment interaction (GEI *p* value) and genetic correlation for line values across pairs of test environments (*r_g_*) for traits of 306 pea lines belonging to three connected RIL populations.

Genetic correlation	Lodi 2013–14 vs. Lodi 2014–15		Lodi 2013–14 vs. Perugia 2013–14	
Trait	GEI *p* value	*r_g_*	GEI *p* value	*r_g_*
Yield (t/ha)	**	0.35^**^	**	0.79^**^
Protein content (%)	**	0.73^**^	**	0.92^**^
Protein yield (t/ha)	**	0.34^**^	**	0.80^**^

** *p value of GEI significant at p < 0.01, or r_g_ different from zero at p < 0.01*.

Grain yield and protein content exhibited a slightly positive genetic correlation in all environments, which reached *p* < 0.05 significance only in Lodi 2014–2015 (Table 5). Line protein yield was overwhelmingly affected by grain yield, on the ground of phenotypic correlations of protein yield with its two component traits (Table 5).

**Table 5:** Genetic correlation between grain yield (GY) and grain protein content (GPC), and phenotypic correlation between protein yield per unit area (PY) and its two component traits (GY and GPC), for 306 pea lines belonging to three connected RIL populations.

Environment	Genetic correlation ± SE	Phenotypic correlation	
	GY - GPC	PY - GY	PY - GPC
Lodi 2013–14	0.12 ± 0.08^NS^	0.98^**^	0.30^**^
Lodi 2014–15	0.18 ± 0.07^*^	0.99^**^	0.24^**^
Perugia 2013–14	0.14 ± 0.08^NS^	0.99^**^	0.29^**^

* *p < 0.01;*

** *p < 0.05*.

NS *Not significant (p > 0.05)*.

### Assessment of Genomic Selection Models and Intra- and Inter-population Genomic Predictions

Next generation sequencing produced, on average, 551,210 reads per sample. The number of polymorphic SNP markers was severely affected by the allowed mpm and mps values (Supplementary Table 3). The first GS scenario, represented by intra-population, inter-environment prediction, was employed to determine the most convenient model and model configuration to adopt for both prediction scenarios. Thresholds of mpm below 0.05 always implied too few polymorphic markers (<500; Supplementary Table 3). Therefore, we tested models with mpm values in the range 0.05–0.30 combined with mps thresholds between 0.10 and 0.50, which produced a number of polymorphic SNPs ranging from 2,297 to 30,464 (Supplementary Table 3). Just slight differences in predictive ability were reported for the three traits for these combinations of mpm and mps, observing a consistent trend across environments towards lower GS predictive ability only for the combination of mps = 0.1 and mpm = 0.3 for grain protein content (Supplementary Figure 1). We selected for subsequent analyses the thresholds mpm = 0.2 and mps = 0.25, which ensured a good compromise between model predictive ability and number of samples in the dataset (Supplementary Figure 1 and Supplementary Table 3).

The four GS models tended to perform very similarly for intra-population, inter-environment predictive ability of the target traits averaged across validation environments and RIL populations, albeit with a very slight overall advantage of rrBLUP, which was selected for subsequent analyses (Table 6). Mean predictive ability in this scenario (which assumed two environments for model construction) was moderately high for protein content (*r* = 0.53), and moderate for grain and protein yield (*r* = 0.40 and *r* = 0.41, respectively; Table 6). Intra-population, inter-environment predictions for the single validation environments did not differ markedly depending on the pair of GS model training environments (Table 7). They indicated somewhat greater difficulty of predicting grain and protein yield in Perugia by GS model training based on data of two cropping seasons in Lodi, as well as somewhat greater difficulty of predicting grain protein content in Lodi 2014–2015 based on model training in the other two environments (Table 7).

**Table 6:** Predictive ability for three traits of four genomic selection models in the intra-population, inter-environment scenario obtained by using two environments for model training and one for validation.

Model^a^	Grain yield	Protein content	Protein yield
Ridge regression BLUP	0.403	0.529	0.406
Bayesian C	0.395	0.530	0.397
Bayesian A	0.394	0.531	0.396
Bayesian Lasso	0.398	0.524	0.397

Results averaged across three connected RIL populations and all possible validation environments.*aValues of individual analyses averaged across results of a 10-fold stratified cross-validation scheme with 10 repetitions, relative to a total number of 306 lines*.

**Table 7:** Intra-population and inter-population inter-environment predictive ability for three pea traits obtained by Ridge regression BLUP modelling using two environments for model training and one for validation and, for inter-population predictions, one RIL population for model training aimed to predictions for the other populations.

Trait	Intra-population inter-environment^a^				Inter-population inter-environment			
	Validation environment				RIL population used for training			
	Lodi 2013–14	Lodi 2014–15	Perugia 2013–14	Mean	A × I	K × A	K × I	Mean
Yield (t/ha)	0.39	0.45	0.36	0.40	0.08	0.28	0.27	0.21
Protein content (%)	0.60	0.45	0.53	0.53	0.27	0.21	0.32	0.27
Protein yield (t/ha)	0.40	0.46	0.36	0.41	0.08	0.25	0.27	0.20

Results relative to three RIL populations derived from connected crosses (A × I, 102 lines; K × A, 100 lines; K × I, 104 lines) averaged across all possible validation environments.*aAveraged across results for each of three RIL populations based on a 10-fold stratified cross-validation scheme with 10 repetitions*.

Adopting inter-population instead of intra-population, inter-environment predictions implied an average decrease of predictive ability around 50% for all traits (Table 7). Model training on A × I led to distinctly inferior predictions for grain and protein yield (Table 7). Inter-population predictions for grain protein content were not only higher on average, but also less affected by the choice of the RIL population for GS model training compared with those for the other two traits (Table 7).

### Comparison of Genomic vs. Phenotypic Selection

Based on correlation results in Table 8, the ability of line phenotypic data averaged across two environments to predict line phenotypic data in a third environment was similar to that of GS-modeled data trained in two environments for prediction in a third environment. In particular, a modest advantage was displayed by phenotypic data for protein content and by GS-modeled data for grain and protein yield.

The predicted efficiency of GS relative to PS was heavily influenced by the GS prediction scenario (intra- or inter-population inter-environment prediction) and by the assumed type and cycle duration of PS (selection performed in two locations during the same year, or in the same or a different location across 2 years). The predicted advantage of GS relative to PS was particularly high (over 4-fold efficiency) when assuming intra-population prediction and a two-year PS cycle, was about nil when assuming inter-population predictions and a one-year PS cycle, and was sizeable (over two-fold efficiency) in the other cases (Table 9).

**Table 8:** Correlation of phenotypic data or genomic selection (GS)-modelled data based on two test environments with data in a third (validation) environment, averaging results for all pairs test environments.

Trait	Phenotypic data	Data predicted by GS
Yield (t/ha)	0.46	0.48
Protein content (%)	0.75	0.70
Protein yield (t/ha)	0.49	0.51

Values averaged across results for each of three connected RIL populations and all possible validation environments.

**Table 9:** Ratio of genomic selection (GS) to phenotypic selection (PS) efficiency for protein yield based on predicted genetic gains per unit time for similar evaluation costs assuming two environments for PS and for generation of phenotyping data for intra-population and inter-population GS scenarios.

Trait	*H_C_*	GS_A_*r_Ac_*	GS_A_/PS efficiency ratio		GS_B_*r_Ac_*	GS_B_/PS efficiency ratio	
			*t_P_* = 1	*t_P_* = 2		*t_P_* = 1	*t_P_* = 2
Protein yield (t/ha)	0.676	0.511	2.192	4.383	0.252	1.084	2.167

*H_C_* is the square root of the broad-sense heritability on a genotype mean basis; *r_Ac_* is the GS predictive accuracy for intra-population (GS_A_) or inter-population (GS_B_) prediction scenarios; *t_P_* is the duration of one cycle of PS (one or two years). Efficiency ratios averaged across separate analyses for all possible validation environments and three connected RIL populations.

### Genome-Wide Association Study

**Figure 1:**
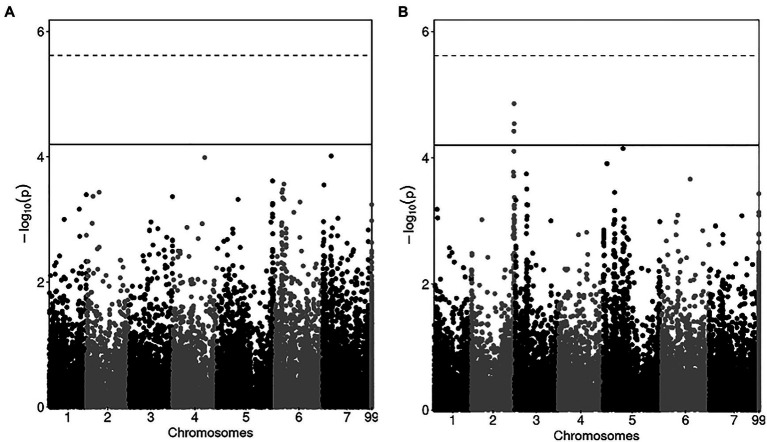
Manhattan plots showing the association score of SNP markers with grain yield **(A)** and grain protein content **(B)** along pea chromosomes in a GWAS based on 306 lines belonging to three connected RIL populations. The dashed line represents the Bonferroni correction threshold, while the solid line represents False Discovery Rate threshold in **(A)**, and the threshold employed to select significantly associated markers in **(B)**.

The results of the GWAS performed on stratified data of the three RIL populations for grain yield and protein content are summarized by the Manhattan plots in Figure 1, which report the association scores of the SNP markers with the two traits along the pea genome. For both traits, the plots indicated many regions in the genome featuring a slight association pattern, as expected for complex polygenic traits. Trait-marker association inspection according to the False Discovery Rate threshold detected no SNP marker significantly associated with grain yield, and three markers placed on chromosome 2 significantly associated with grain protein content (Figure 1; Supplementary Table 4). However, none of these markers achieved the Bonferroni correction threshold of significance (Figure 1). The three markers featuring a possible association with protein content displayed linkage disequilibrium ranging from 0.20 to 0.67, suggesting that the actual number of QTL they refer to may be less than three. The phenotypic variance that they explained ranged from 5.5 to 6.1% (Supplementary Table 4), which further confirmed the definite polygenic control of protein content. The genes of known function immediately proximal to these markers encode a glycosyl hydrolase of family 9 (Psat2g183720) and a cytochrome C signature protein (Psat2g187160).

## Discussion

This study, which adds to results for grain yield and other traits of pea genotypes in Italian environments by Annicchiarico et al. (2019), showed that grain protein content is less challenging than grain yield for phenotypic or genome-enabled selection. This conclusion descends from lower influence of GEI (Tables 3 and 4), which simplifies PS and reduces the need for multi-environment phenotyping aimed at GS model training, and greater GS predictive ability of this trait relative to grain yield. Another encouraging result for pea protein content improvement was the absence of genetically-based trade-offs between this trait and grain yield. This result was highlighted by genetic correlations for separate test environments and was confirmed by different genomic regions controlling these traits in the GWAS. As anticipated, the absence of a negative relationship between these traits was suggested by some earlier studies (Cousin et al., 1985; Klein et al., 2020; Bărbieru, 2021), but not by others (Tar’an et al., 2004; Krajewski et al., 2012) based on phenotypic correlations, while no earlier assessment of genetic correlation is apparently available.

The range of phenotypic variation for grain protein content, close to 5%, was intermediate relative to values reported for inbred lines or cultivars in earlier studies (Cousin et al., 1985; Tar’an et al., 2004; Burstin et al., 2007; Jha et al., 2015). It was much lower than that in Ferrari et al. (2016) for material belonging to the same genetic base analyzed in just one test environment, a difference that may partly be explained by the GEI tendency to decrease the range of variation of line values averaged across environments (as in the current study) compared to line variation in individual environments. The current RIL populations, obtained by crosses between elite varieties selected on the ground of grain yield rather than protein content, are likely to be representative of much material generated by ordinary pea breeding programs. This view is supported by the high agronomic value for grain and protein yield exhibited by several breeding lines relative to a locally-elite commercial cultivar such as Spacial. The occurrence of much greater genetic variation within RIL populations than among RIL populations for all target traits according to estimated variance components emphasized the practical importance of within-population selection, as currently focused by GS predictions, to increase the probability to select genotypes featuring rare recombination events among several favorable alleles.

Pea protein yield per unit area, which seemingly is the main target trait for crop use as a high-protein feedstuff, was affected by grain yield to a much greater extent than by grain protein content. Accordingly, the results for protein yield paralleled those for grain yield with respect to genetic variation (both as *CV_g_* value and relative extent of inter- and intra-population variation: Tables 2 and 3), GEI extent and pattern (Tables 3 and 4), and quality of genome-enabled predictions (Tables 6 and 7). The indication of greater size of genotype × year interaction compared with genotype × location interaction that emerged for these traits in the target region suffered from the limited number of test years and locations but agreed with grain yield results from two studies based on a larger sample of environments (Annicchiarico and Iannucci, 2008; Pecetti et al., 2019). These reports highlighted the relationship of genotype × year interaction for grain yield with year-to-year variation for extent of low winter temperatures, a relationship that held true also for this data set, as reported in detail in Annicchiarico et al. (2019). This GEI pattern justified the selection for wide adaptation across northern and central Italy that was devised for assessing PS or GS strategies for protein yield and the consideration, in this context, also of a two-year selection scenario for PS beside a one-year scenario. The increasing year-to-year climatic variability occurring in the target region as a consequence of climate change is enhancing the importance of GEI variance components relative the interaction of genotype with year relative to the genotype × location variance component in another autumn-sown rainfed crop such as durum wheat (Annicchiarico, 2020).

GS results were produced by the rrBLUP model, but its predictive ability advantage over three Bayesian models was negligible. Accordingly, only slight differences in predictive ability among most tested genomic prediction models were reported in earlier pea studies for grain yield or other traits (Burstin et al., 2015; Annicchiarico et al., 2017, 2019). The similar correlation with phenotypic data in an independent environment exhibited by GS-modelled data compared to the phenotypic data they were based upon (Table 8) was reported as well in previous studies on pea (Tayeh et al., 2015; Annicchiarico et al., 2019). This result suggests that the disadvantage of partly unaccounted genetic variation by GS models may be counterbalanced by the ability of these models to reduce the noise of phenotypic data.

A major finding of this study is the moderately high genome-enabled intra-population, inter-environment predictive ability for grain protein content (*r* = 0.53) and the moderate predictive ability for crop protein yield (*r* = 0.41). The higher predictive ability reported here for grain yield relative to Annicchiarico et al. (2019) was mainly due to the greater number of environments employed in this study for GS model training (two vs. one), without ruling out the effect of the different SNP calling procedure adopted by this study (pea genome-based) relative to the earlier one (mock genome-based). The greater inter-environment predictive ability of protein content relative to grain or protein yield can be attributed to its higher heritability over environments as determined by greater variance of purely genetic effects relative to GEI effects. Two environments (albeit not necessarily in different years) for GS model training, which were deemed necessary because of the possibly large GEI for grain and protein yield, produced GS predictions not only moderately accurate, but also limitedly affected by the specific pair of environments adopted for GS model training. Greater predictive ability for the target traits may have arisen from greater number of test environments used for GS model training. While possibly underestimating the ability of GS modelling to predict trait variation, our assumption of two test environments for model training reflected the need for breeding programs to limit the investment in phenotyping work for a cost-efficient application of GS, also considering that other phenotyping work on different training sets may be needed for GS model definition targeted to completely unrelated breeding populations.

The decrease of genome-enabled predictive ability passing from the intra-population to the inter-population scenario for inter-environment predictions approached 50% for all traits, but its value varied remarkably for grain and protein yield depending on the RIL population used for GS model training. The distinctly inferior ability of A × I when used as a training set to predict grain and protein yield of the other RIL populations agreed with previous results for grain yield under severe drought and for onset of flowering reported for the same materials in an earlier study by Annicchiarico et al. (2017) that showed, in addition, higher number of polymorphic markers shared by K × A and K × I than by each of them and A × I. The poor ability of A × I as a training set for inter-population prediction of production traits may largely be due to the fact that A × I excluded the genome of the Australian cultivar, whose genetic dissimilarity from either European cultivar was definitely greater than that between the two European cultivars according to Nei’s (1972) genetic distance values reported in Annicchiarico et al. (2019). Hence, wider genetic diversity of the founding parents possibly assessed by *ad-hoc* work prior to selection of RIL populations for GS model training may enhance the predictive ability of a RIL population for other populations having one common parent. Results for grain protein content (where the A × I had intermediate predictive ability for the other RIL populations) indicated, however, that this is not necessarily the case.

The GWAS aimed mainly at deepening our knowledge of the genetic control of pea grain yield and protein content. Incidentally, this analysis allowed for greater statistical power for linkage detection relative to ordinary genetic linkage mapping analysis performed separately for each of the three RIL populations, when comparing the two methods according to Wang and Xu (2019) on the basis of their respective genotype sample sizes and a proportion of phenotypic variation explained by each QTL in the range 1–10% (data not reported). The results of the GWAS confirmed the definite polygenic control of the two traits, thereby supporting the interest of developing GS models for both of them and/or protein yield rather than focusing on the search of associated markers for MAS. Our detection of putative QTL for grain protein content on chromosome 2 agrees with earlier findings from various reports (Burstin et al., 2007; Bourion et al., 2010; Klein et al., 2014; Gali et al., 2018). In particular, Klein et al. (2014) reported three QTL in the same region of chromosome 2 containing the loci detected in this study. The first associated region found on chromosome 2 includes the gene Psat2g185440, identified as a candidate transcription factor for the control of seed vicilin content in pea (Le Signor et al., 2017), as well as the gene Psat2g185800 showing high sequence similarity with three *M. truncatula* genes (Medtr5g009160, Medtr8g096880, Medtr5g009160) involved in the synthesis of symbiosome membrane components (Santi et al., 2017).

This study provided an unprecedented comparison of GS vs. PS for protein yield improvement in pea. Its results, based on predicted gains per unit time and similar evaluation costs, indicated an advantage of GS when model training included the target RIL population over all PS scenarios, as well as an advantage of GS when model training was based on a RIL population sharing one parent with the target population and PS stretched over two cropping years. Efficiency ratios of GS vs. PS were affected by our estimates of selection costs per trait and/or genotype, which were somewhat higher for GS than those in Annicchiarico et al. (2019). However, our results are encouraging for GS, particularly when GS model training includes material of the RIL population targeted by selection and GS is envisaged as an alternative to multi-year PS. GS model training using a two-year data set can be recommended for Italy because of the GEI size across years for grain yield. A crucial confirmation of the advantage of GS over PS for pea protein yield improvement will be provided by future research work comparing these selection strategies in terms of actual yield gains.

## Data Availability Statement

The datasets presented in this study can be found in online repositories. The names of the repository/repositories and accession number(s) can be found at: https://www.ncbi.nlm.nih.gov/, https://www.ncbi.nlm.nih.gov/bioproject/PRJNA727737/.

## Author Contributions

MC was responsible for the analysis of phenotyping data, wrote the paper, and contributed to the analysis of genotyping data. NN was responsible for the analysis of genotyping data. BF was responsible for DNA sampling and collection of protein content data. LP, LR, and MR were responsible for field trials and collection of grain yield data. GC developed NIRS predictions. DC contributed to the analysis of phenotyping data. AM supervised the work by MC, which was part of a Ph.D. thesis. PA was responsible for funding acquisition, devised the research work, and supervised the manuscript drafting. All authors contributed to the article and approved the submitted version.

## Funding

This research was performed within the project “Genomic selection for yield, drought tolerance and protein content of grain and forage legumes (GENLEG)” funded by the Italian Ministry of Agricultural, Food and Forestry Policies (MiPAAF). Field experiments were performed within the project “Coordinating Organic Plant Breeding Activities for Diversity (COBRA)” also funded by MiPAAF.

## Conflict of Interest

The authors declare that the research was conducted in the absence of any commercial or financial relationships that could be construed as a potential conflict of interest.

## Publisher’s Note

All claims expressed in this article are solely those of the authors and do not necessarily represent those of their affiliated organizations, or those of the publisher, the editors and the reviewers. Any product that may be evaluated in this article, or claim that may be made by its manufacturer, is not guaranteed or endorsed by the publisher.

## Footnotes

^1^ https://urgi.versailles.inra.fr/download/pea/
