# Supplementary material for: Pea Grain Protein Content Across Italian Environments: Genetic Relationship With Grain Yield, and Opportunities for Genome-Enabled Selection for Protein Yield
Source: Front Plant Sci. 2022 Jan 3;12:718713. doi: 10.3389/fpls.2021.718713 (PMC8761899; doi:10.3389/fpls.2021.718713)
Supplement: Supplementary file 1 [file Table_1.DOCX]

**Supplementary Table 1 |** Climate, soil, and long-term climate characteristics of three pea test environments.

| **Item** | **Lodi 2013-14** | **Lodi 2014-15** | **Perugia 2013-14** | **Lodi long-term** | **Perugia long-term** |
| --- | --- | --- | --- | --- | --- |
| Crop management system | Organic | Conventional | Organic | - | - |
| Rainfall, Jan.-Mar. (mm) | 343 | 198 | 280 | 161 | 177 |
| Rainfall, Apr.-May (mm) | 122 | 147 | 179 | 154 | 142 |
| Absolute minimum daily temp. (°C) | –5.7 | –11.6 | –3.6 | –7.7 | –5.0 |
| Mean of max. daily temp., May (°C) | 23.2 | 23.9 | 23.4 | 21.8 | 23.0 |
| Soil texture^A^ | Silt-loam | Sandy-loam | Silty-clay-loam | - | - |
| Soil pH | 7.9 | 6.3 | 7.6 | - | - |

*According to FAO (2006) Guidelines for soil description, 4^th^. Rome: Food and Agricultural Organization.*
