# Supplementary material for: Pea Grain Protein Content Across Italian Environments: Genetic Relationship With Grain Yield, and Opportunities for Genome-Enabled Selection for Protein Yield
Source: Front Plant Sci. 2022 Jan 3;12:718713. doi: 10.3389/fpls.2021.718713 (PMC8761899; doi:10.3389/fpls.2021.718713)
Supplement: Supplementary file 2 [file Table_2.DOCX]

**Supplementary Table 2 |** Mean value of parental lines of three connected RIL populations and of the cultivar Spacial for three pea traits in three test environments.

| **Trait** | **Environment** | **Mean value** | | | |
| --- | --- | --- | --- | --- | --- |
|  |  | **Attika** | **Kaspa** | **Isard** | **Spacial** |
| Yield (t/ha) | Lodi 2013-2014 | 4.97 b | 7.16 a | 6.36 ab | 7.82 a |
|  | Lodi 2014-2015 | 1.34 c | 2.14 c | 6.39 a | 3.66 b |
|  | Perugia 2013-2014 | 2.13 c | 3.34 ab | 2.59 bc | 3.70 a |
| Protein content (%) | Lodi 2013-2014 | 23.68 c | 26.82 a | 24.30 bc | 24.87 b |
|  | Lodi 2014-2015 | 22.71 b | 23.54 a | 21.90 c | 21.68 c |
|  | Perugia 2013-2014 | 22.89 c | 26.08 a | 23.04 c | 24.16 b |
| Protein yield (t/ha) | Lodi 2013-2014 | 1.18 c | 1.92 a | 1.54 b | 1.95 a |
|  | Lodi 2014-2015 | 0.30 c | 0.50 bc | 1.40 a | 0.80 b |
|  | Perugia 2013-2014 | 0.49 b | 0.87 a | 0.60 b | 0.90 a |

*Row means followed by different letter differ at* P *< 0.05.*
