# Supplementary material for: Pea Grain Protein Content Across Italian Environments: Genetic Relationship With Grain Yield, and Opportunities for Genome-Enabled Selection for Protein Yield
Source: Front Plant Sci. 2022 Jan 3;12:718713. doi: 10.3389/fpls.2021.718713 (PMC8761899; doi:10.3389/fpls.2021.718713)
Supplement: Supplementary file 3 [file Table_3.DOCX]

**Supplementary Table 3 |** Combinations of missing per marker (mpm) and missing per sample (mps) thresholds and corresponding number of markers and samples retained in the dataset, and of samples removed from the dataset. Data for intra-population, inter-environment genomic selection scenario obtained by Ridge regression BLUP model.

| **Mpm** | **Mps** | **Number of markers** | **Number of samples** | **Samples removed** |
| --- | --- | --- | --- | --- |
| 0.01 | 0.5 | 21 | 306 | 2 |
| 0.01 | 0.25 | 21 | 306 | 2 |
| 0.01 | 0.1 | 21 | 303 | 5 |
| 0.03 | 0.5 | 324 | 302 | 6 |
| 0.03 | 0.25 | 324 | 297 | 11 |
| 0.03 | 0.1 | 324 | 289 | 19 |
| 0.05 | 0.5 | 2297 | 297 | 11 |
| 0.05 | 0.25 | 2297 | 290 | 18 |
| 0.05 | 0.1 | 2297 | 281 | 27 |
| 0.1 | 0.5 | 10887 | 289 | 19 |
| 0.1 | 0.25 | 10887 | 280 | 28 |
| 0.1 | 0.1 | 10887 | 275 | 33 |
| 0.15 | 0.5 | 16757 | 286 | 22 |
| 0.15 | 0.25 | 16757 | 279 | 29 |
| 0.15 | 0.1 | 16757 | 265 | 43 |
| 0.2 | 0.5 | 21383 | 286 | 22 |
| 0.2 | 0.25 | 21383 | 277 | 31 |
| 0.2 | 0.1 | 21383 | 253 | 55 |
| 0.3 | 0.5 | 30464 | 285 | 23 |
| 0.3 | 0.25 | 30464 | 270 | 38 |
| 0.3 | 0.1 | 30464 | 188 | 120 |
