# Supplementary material for: Pea Grain Protein Content Across Italian Environments: Genetic Relationship With Grain Yield, and Opportunities for Genome-Enabled Selection for Protein Yield
Source: Front Plant Sci. 2022 Jan 3;12:718713. doi: 10.3389/fpls.2021.718713 (PMC8761899; doi:10.3389/fpls.2021.718713)
Supplement: Supplementary file 4 [file Table_4.DOCX]

**Supplementary Table 4 |** Chromosome, position, association significance (-log(*P*)), and proportion of explained variance of SNP markers significantly associated with protein content in a GWAS based on 306 pea lines from three connected RIL populations.

| **Trait** | **SNP** | **Chromosome** | **Position** | **-log(*P*)** | **Explained variance** |
| --- | --- | --- | --- | --- | --- |
| Protein content | chr2LG1_422159521 | 2 | 4.22E+08 | 4.42 | 5.5% |
| Protein content | chr2LG1_423695805 | 2 | 4.24E+08 | 4.86 | 6.1% |
| Protein content | chr2LG1_424873125 | 2 | 4.25E+08 | 4.54 | 5.7% |
