# Supplementary figures and images for: Pea Grain Protein Content Across Italian Environments: Genetic Relationship With Grain Yield, and Opportunities for Genome-Enabled Selection for Protein Yield
Source: Front Plant Sci. 2022 Jan 3;12:718713. doi: 10.3389/fpls.2021.718713 (PMC8761899; doi:10.3389/fpls.2021.718713)

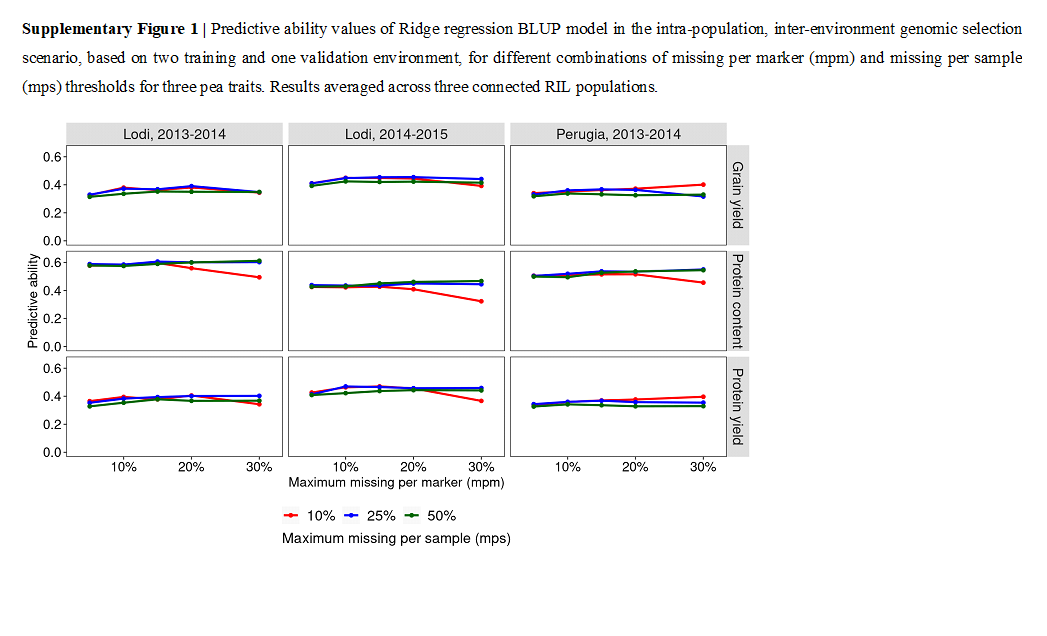

Supplement: Supplementary file 5 [file Image_1.TIF]

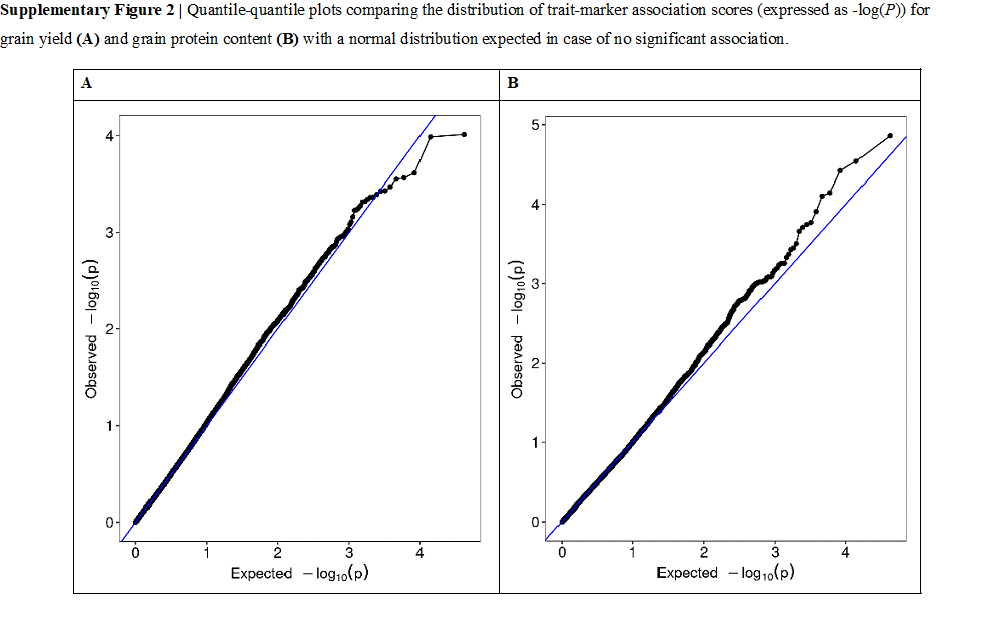

Supplement: Supplementary file 6 [file Image_2.tif]
